# Abscopal Effect in a Stage IV Melanoma Patient who Progressed on Pembrolizumab

**DOI:** 10.7759/cureus.2238

**Published:** 2018-02-27

**Authors:** James M Tsui, Catalin Mihalcioiu, Fabio L Cury

**Affiliations:** 1 Department of Oncology, Division of Radiation Oncology, Cedars Cancer Centre / McGill University Health Centre, Montreal; 2 Department of Oncology, Division of Medical Oncology, Cedars Cancer Centre / McGill University Health Centre, Montreal

**Keywords:** melanoma, abscopal effect, immunotherapy, radiotherapy, palliative

## Abstract

In this case report, we present the clinical course of a woman with locally advanced mucosal melanoma of the oral cavity. She was initially treated with surgery with adjuvant local radiation of 50 Gy in 20 fractions. She quickly relapsed with an aggressive regional recurrence of the disease on the neck and with numerous pulmonary metastases. Immunotherapy with pembrolizumab was started, with initial good response and reduction in the size of the lesion in the neck. The regression, however, was short-lived, as the mass quickly grew at a remarkable rate and the lung lesions progressed significantly. Palliative local radiation of 24 Gy in three fractions delivered at days zero, seven, and 21 to the neck mass was eventually given with the goal to alleviate symptoms. An immediate tumor regression was observed after the first fraction of radiotherapy. Remarkably, the lung lesions had also started regressing following radiation. We believe this to be a case of abscopal effect witnessed during the delivery of radiotherapy. A review of the recent literature is also presented here.

## Introduction

The abscopal effect, first reported by Mole in 1953, is a phenomenon whereby irradiation of a tumor lesion triggers spontaneous regression of metastatic disease at a distant site [1]. Since then, animal models have provided insight into the underlying physiological mechanism of the abscopal effect, which is thought to be immune-mediated; irradiation of a lesion causes increased tumor antigen release and presentation to T-cell, subsequently enhancing the immunological response. This phenomenon appears to be synergized with the presence of immunotherapeutic agents [2]. With the increasing use of ipilimumab for treatment of metastatic melanoma following Food and Drug Administration (FDA) approval in 2011, several reports have emerged describing cases of abscopal effect [2-4]. Here, we report a case of metastatic disease resolution following local radiation therapy on a patient with metastatic melanoma who initially progressed on pembrolizumab.

## Case presentation

A 65-year-old woman presented with metastatic mucosal melanoma with a history of dyslipidemia and using proton pump inhibitor for gastroesophageal reflux. She had a remote tonsillectomy in the 1970s and was otherwise healthy. At the beginning of 2015, she noticed a discoloration on the right buccal mucosa and eventually sought medical attention. When first examined in oncology, in June 2015, the lesion was extending to the soft and hard palate. A punch biopsy of the lesion revealed that it was malignant melanoma (immunostains for Mart1 and HMB45 were positive). Initially, the tumor was not visualized on contrasted computerized tomography (CT) of the neck, nor on magnetic resonance imaging (MRI) with gadolinium. Investigation of metastatic disease including CT of the chest and abdomen were negative. The case was presented to the Tumor Board, and the consensus was for a surgical approach.

In August 2015, she underwent right partial maxillectomy and partial marginal mandibulectomy with midline mandibulotomy and free vascular flap. She also had ipsilateral selective neck dissection of levels I to IV, as well as a tracheostomy. Pathology revealed poorly differentiated malignant melanoma of the maxillary gingiva and hard palate, measuring 2 cm in largest dimension, with an invasion of the maxillary bone at the last molar, as well as satellitosis (more than five atypical mitoses were observed at high power field; mutation for BRAF was negative). The resection margins were clear; the closest margin was to the anterior tonsillar pillar at 10 mm. Out of the 25 lymph nodes dissected, one harbored a focus of melanoma measuring 1 mm, without extra-nodal extension. The pathological staging was thus pT4a pN1.

**Figure 1:**
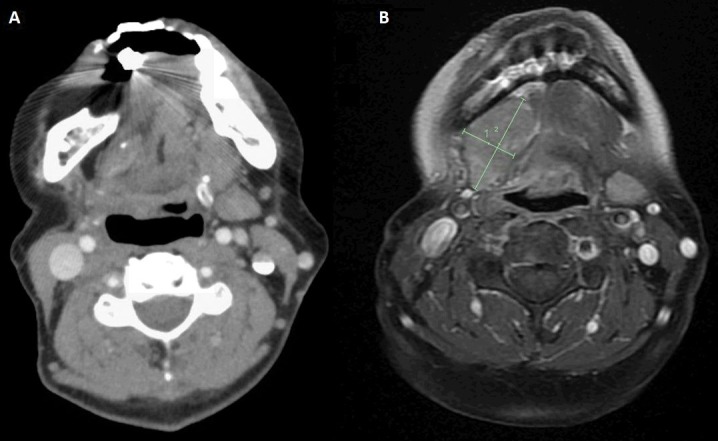
Diagnostic imaging (A) Computerized tomography (CT) scan of the neck with contrast performed in May 2016. Postoperative changes of right-sided partial maxillectomy and marginal mandibulectomy with flap reconstruction can be seen. There was a tissue with intermediate density in the infratemporal fossa, and partially surrounding the right pterygoid process. At the time of the exam, the radiologist could not conclude whether this was scar tissue or tumor recurrence. (B) Magnetic resonance imaging (MRI) scan of the neck with contrast performed in June 2016. The lesion was seen along the right side of the floor of the mouth measuring 1.9 x 3.5 x 3.9 cm in maximum dimensions compared to 1.4 x 2.5 x 2.3 cm along the same dimensions previously. The lesion was inseparable from the right mylohyoid muscle and of the remainder of the floor of the mouth muscles. No definite invasion or significant abnormality of the adjacent portion of the mandible was seen.

**Figure 2:**
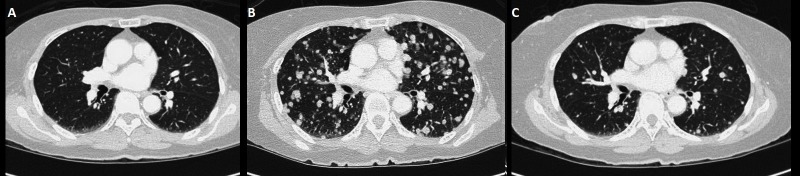
Distal response to radiation (A) Computed tomography (CT) of the chest in August 2016, showing diffuse pulmonary nodules. (B) Repeat CT of the chest in October 2016 showing remarkable interval progression of the pulmonary nodules. There was no mediastinal lymphadenopathy. (C) Follow-up CT of the chest in November 2016 following completion of palliative radiation to the neck; significant decrease in the size and number of the bilateral pulmonary nodules can be seen.

She was then seen by radiation oncology and underwent a course of adjuvant therapy of 50 Gy in 20 fractions between October to November 2015. The treatment was delivered using intensity modulated radiotherapy (IMRT) to the site of the resected primary tumor, and it was well tolerated with moderate acute side effects - grade 2 mucositis, and grade 1 dermatitis. Clinically she recovered well during follow-up, with difficulty masticating food due to her teeth not aligning following surgery, so she ate mostly pureed food and diet supplementation via percutaneous endoscopic gastrostomy (PEG) tube. A follow-up CT scan of the neck in May 2016 revealed a new soft tissue density at the floor of the mouth, which was outside the irradiated field. No evidence of lymphadenopathy was seen (Figure 1A). The mass grew rather quickly, and in June 2016, a new right neck mass was seen on level I/ II. Fine needle aspiration of the mass showed the presence of metastatic melanoma. An MRI scan of the neck revealed a rapidly progressing soft tissue lesion along the right side of the floor of the mouth (Figure 1B). Further metastatic work-up from June found new multiple sub-centimetric lung nodules bilaterally. She did not have significant lymphadenopathy, and CT scan of the abdomen revealed no metastatic disease. Positron emission tomography (PET)-CT scan performed in July 2016 showed increased metabolic activity to the lesion on the floor of the mouth, and a mildly hypermetabolic small left cervical lymph node at level two that was thought to be more likely reactive. The pulmonary nodules were too small to be resolved metabolically in the PET study, but a repeat CT scan of the chest in August showed pulmonary nodules increasing in size (Figure 2A).

 

She was assessed by medical oncology and was enrolled in an experimental trial comparing epacadostat 100 mg BID for 21 days versus placebo. Both groups received pembrolizumab 200 mg IV (3 mg/kg) every three weeks (protocol MK2475-252). The first cycle started in early August 2016. The tumor initially responded and shrank in size, but this was short-lived; it soon progressed causing symptoms including stiffening of the tongue, pain, increased drooling, as well as dysphagia accompanied with a weight loss of three pounds over four weeks. Due to the progression of the disease, she was removed from the experimental trial after one cycle but continued to receive pembrolizumab off-study. After a total of four cycles of pembrolizumab, she was referred to radiation oncology for palliative irradiation of the neck for symptom control. At the time of assessment in October 2016, two months following the first cycle of pembrolizumab, she continued to have enlargement of the neck tumor causing increasing dysphagia. There was no ulceration, but the mass involved the skin and extended from the mandible down to the clavicle with small satellite nodes next to it. A palliative course of radiation of 24 Gy in three fractions on days zero, seven, and 21 was delivered using IMRT.

**Figure 3:**
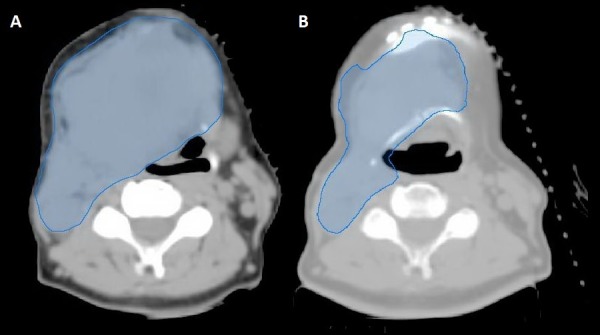
Computed tomography (CT) simulation images (A) CT-simulation performed on October 13th, 2016. (B) Repeat CT-simulation, performed on November 7th, 2016.

Figure 3A shows the CT simulation performed on October 13th, 2016. The imaging showed the progression of the disease as compared to the images from June 2016, with the tumor now causing mass effect and putting the patient at risk of obstructing the airway. She received the first radiation treatment on October 19th, to which she responded almost immediately with a clinical reduction in tumor size of approximately 20% only after a week post-radiation. She received the second radiation treatment on October 26th. For the third fraction, she underwent a new CT-sim and re-plan because of the reduction in the tumor size (Figure 3B). The most surprising response to the course of irradiation to the neck area was the regression of the lung lesions.

**Figure 4:**
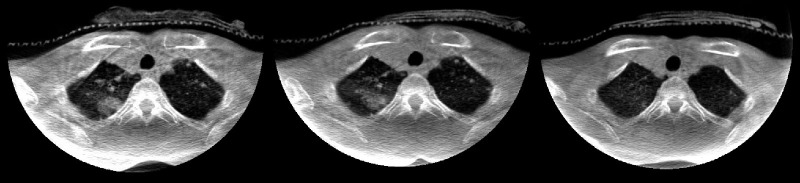
Cone beam computed tomography (CBCT) images CBCT used for patient radiation treatment positioning purposes (not diagnostic). The images are obtained from CBCT scans prior to every treatment. From left to right, images were taken on October 19th, 26th, and November 9th, 2016 respectively.

Before commencement of radiation, it was noted by medical oncology in October 2016 (ten weeks and five days after the first cycle of immunotherapy) that she was having increasing shortness of breath. The fifth cycle of immunotherapy was held for this reason. CT imaging of the chest in October 2016 (nine days after the first fraction, and two days after the second fraction of radiation) showed interval progression of the diffuse bilateral pulmonary metastases when compared to a previous CT-scan (Figure 2B). However, by the time of imaging, the dyspnea had already resolved. Imaging of the chest was not done immediately before the commencement of irradiation when she was most symptomatic. She received the third fraction of radiation on November 9th, 2016. Repeat CT scan of the chest performed on November 21st, 2016 showed that the lesions previously seen were remarkably reduced both in size and in numbers (Figure 2C). The cone beam CT done for positioning purposes also provided valuable information about the evolution of the lung disease. Although only the apices of the lungs were captured in the images, regression of lung lesions in size and number following the first fraction of irradiation of the local tumor in the neck was observed (Figure 4).

**Figure 5:**
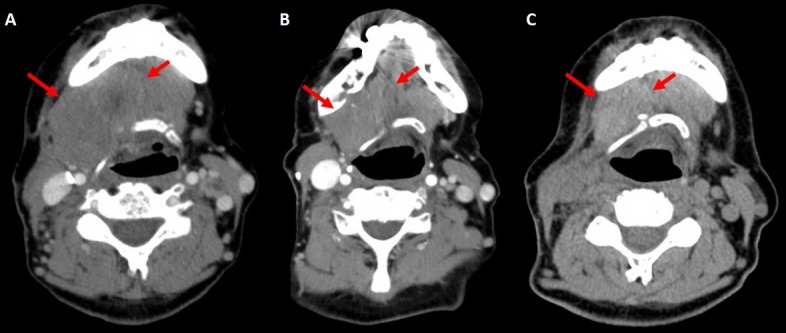
Local response to radiation (A) Computed tomography (CT) of the neck on October 28th, 2016. (B) Same performed on November 21st, 2016 after completion of three fractions of palliative irradiation, showing a reduction in the size of the main lesion as well as a reduction in size and numbers of adenopathy. (C) CT scan of the neck on March 28th, 2017. Unfortunately, IV-contrast was not used, so accurate assessment was difficult. However, the bulk of the disease had reduced in size.

The neck mass had also decreased significantly. Figure 5A shows a CT imaging of the neck performed in October 2016, two days after the second fraction of radiation. Compared to June 2016, there was an interval growth of the large neck mass with increased in size of adenopathy. However, it should be noted again that no baseline chest imaging was performed before the commencement of palliative irradiation. Clinically, the tumor mass was responding to radiation. Repeat imaging performed three weeks following completion of the three fractions of radiation therapy showed a significant reduction in the size of the main neck mass as well as a reduction in size and numbers of neck adenopathy (Figure 5B). Figure 5C is the latest CT imaging in March 2017. Unfortunately, the study was performed without contrast, so accurate assessment of the tumor was difficult. However, the bulk of the disease had diminished in size. PET scan performed in April 2017 revealed significant residual but regressing tumor activity at the right floor of the mouth, (from SUV of 16.8 to 6.1) as compared to July 2016. She continued to do well until July 2017, when two of the lung lesions were found to be growing. They were treated with stereotactic body radiation therapy (SBRT). Her overall disease remained stable, and she continued pembrolizumab for a total of 20 cycles until the end of January 2018, when a repeat CT scan showed progression of disease in the lungs. The plan was to switch her to ipilimumab.

## Discussion

Melanoma is a highly immune-mediated malignancy. The lesions are associated with high infiltrate of immune cells such as melanoma-specific tumor infiltrating lymphocytes, and this has been shown to be a prognostic factor [5]. Considerable efforts have been made over the years to harness the immunogenicity of melanoma for therapy. Indeed, this has shown better results when compared to chemotherapy, but the outcome of metastatic melanoma nevertheless remains dismal.

Recent effort has thus moved to the development of immune-regulating agents. These agents called immune checkpoint inhibitors, target the T-lymphocytes receptors that have an inhibitory effect on their regulatory pathways. The first of such agent approved by the FDA to treat patients with metastatic melanoma was ipilimumab, a CTLA-4 inhibitor that was approved for clinical use in early 2011, and since, several reports of abscopal effect have emerged [2-4]. It appears that Ipilimumab and radiotherapy can confer synergistic activity, and induce enhanced tumor clearance.

Postow and colleagues (2012) reported a case of abscopal effect in a patient with metastatic melanoma who slowly progressed on ipilimumab maintenance treatment [3]. The patient had a cutaneous melanoma that was initially surgically removed. The disease recurred several years later with metastatic lesions to the lung, treated with neoadjuvant chemotherapy followed by lobectomy. During follow-up, she presented with recurrence in the pleural base as well as hilar lymphadenopathy. She was then treated with ipilimumab for a total of four doses, followed by maintenance therapy. Her disease, which was stable during induction therapy, progressed slowly during maintenance therapy. After a year of maintenance therapy, she received a course of palliative radiotherapy consisting of 28.5 Gy in seven fractions only to a lesion in the paraspinal region, following which, she received another dose of ipilimumab. Five months following irradiation, not only had the paraspinal mass regressed, but distant lesions outside the field of radiation, including the hilar lymphadenopathy and splenic lesions, were also found to have regressed significantly. What was interesting about this case report was the pre- and post-radiation immunohistochemical findings. The titer of the antibodies against one of the epitopes on the NY-ESO-1 protein, which was mostly stable throughout the course of immunotherapy before radiation, rose more than 30 times following radiation. The expression of HLA-DR on CD14+ monocytes was also found to have significantly increased after radiotherapy. Conversely, there was a reduction in the quantity of myeloid-derived suppressor cells. When taken together, the authors suggested that radiation may have modulated the host immunological response to enhance activity against tumor cells.

A case series later showed that cases such as the one reported by Postow and colleagues in 2012 [3] may be more common than previously thought [2]. In this retrospective study, the authors first identified 120 patients enrolled in a protocol studying ipilimumab. Of these patients, 21 had disease progression and were subsequently treated with radiation therapy. Among these patients, 11 (52%) were found to not only have a local response, but also a reduction in the size of metastases distant to the irradiated site; this was observed on the CT scan performed 40 days post radiation. The abscopal effect was observed in patients treated with a variety of dose fractionation schemes, ranging from 2 Gy per fraction to 24 Gy in one fraction, and included patients with intra- and extra-cranial sites. The patients with an abscopal response had a significantly longer overall survival compared to the patients with no such effect (22.4 months compared to 8.3 months). What was striking about this study was the large proportion of patients experiencing the abscopal effect, which has been thought to be a rather rare phenomenon [2].

A more recent and larger retrospective analysis also demonstrated that the abscopal effect might not be as rare as initially believed, at least not when radiation is combined with ipilimumab [4]. In this study, 47 consecutive metastatic melanoma patients who were treated with ipilimumab and radiotherapy were analyzed. The radiation dose as well as the treated site varied, including both intra- and extra-cranial sites. The abscopal response was measured by assessing the response to an index lesion that is outside the radiation treatment field. The data were analyzed by courses of radiation as opposed to patients; there were 65 courses of radiation as some patients had more than one course. It was found that in 16 instances (25%), the index lesion decreased in size following radiation. In 11 of which, the index lesion was increasing before radiation. The median survival for their cohort was 28 months. This study also showed that the abscopal effect appears to be associated with a moderately hypofractionated dosage of less than 3 Gy [4]. This may be because a higher dose of hypofractionation (>3 Gy) confers a more effective cell kill, such that even cells responsible for immunomodulation are destroyed, thus lessening the abscopal effect. However, this appears to be at odds with the optimal dose of 8 Gy (x3) in animal models [6]. More research with different fractionation schemes would have to be done in humans to elucidate the question of optimal dose. One major caveat in Chandra et al. (2015) study is that the response was measured with the reduction of index lesion only. It is unclear whether metastatic disease elsewhere in the body was also regressing. It is now apparent that response criteria for cytotoxic agents developed by the World Health Organization (WHO), and later revisited by the Response Evaluation Criteria in Solid Tumors (RECIST) Group, may not accurately capture the disease response to immunotherapy [7].

Wolchok and colleagues (2009) designate this as immune-related response criteria (irRC) [7]. Their work found that there are four distinct response patterns, all of which depended on the total tumor burden. The tumor burden is obtained from the summation of measured size of original index lesions identified before the commencement of immunotherapy and new measurable lesions at the time of assessment. The four response patterns are 1) shrinkage of baseline lesions, 2) stable disease, 3) response after an initial increase in total tumor burden, and 4) response in the presence of new lesions.

It is unclear whether our current case is but an example of pseudo-progression, or delayed response to immunotherapy triggered by radiotherapy. It is conceivable that our patient already had metastatic disease to the lungs that were not yet detectable on CT scan prior to initiation of immunotherapy. What appeared in the lungs shortly after the first cycle of immunotherapy could simply be a manifestation of immune response triggered by pembrolizumab. This certainly fits the third criteria proposed by Wolchok. However, one crucial point to note in our current case is that the main mandibular lesion initially responded to immunotherapy, then progressed while on treatment, thus suggesting treatment failure. It was only following radiation therapy that the patient had systemic control. One plausible explanation is that there was a synergistic effect between radiation therapy and immunotherapy, and either alone would not have been sufficient in providing systemic control.

Another crucial point requiring attention is that our patient was initially enrolled in an experimental protocol, and had received at least one treatment of either epacadostat or placebo. Epacadostat is an inhibitor to indoleamine 2,3 dioxygenase (IDO1), a rate-limiting enzyme that plays a role in the suppression of T cell activity. Unfortunately, we did not obtain permission to unblind the patient from the study she was enrolled in. Thus, it is unclear whether she was in the treatment or placebo arm. However, epacadostat is also an immunomodulator, thus it does not invalidate the possibility of an abscopal effect.

A recent retrospective study of 101 patients with advanced melanoma evaluated the treatment response of ipilimumab alone versus concurrent radiotherapy while on ipilimumab [8]. The primary end-points were overall survival and progression-free survival. The rates of complete and overall responses were also analyzed. The results showed that concurrent radiotherapy with ipilimumab confers significant increase in overall survival and complete response. However, complete response was measured by RECIST criteria. As previously mentioned, it does not accurately assess the response patterns of lesions outside the radiation field, so the abscopal effect in this study was not directly evaluated.

Pembrolizumab has been approved in December 2015 by the FDA to expand the use to metastatic melanoma. Similar to ipilimumab, pembrolizumab suppresses the inhibition of cytotoxic T-cells, but by binding to PD-1 receptors instead of CTLA-4. There is at least one preclinical study that documented the abscopal effect on mice models of melanoma when treated with PD-1 blockade agent and stereotactic ablative radiotherapy [9]. To our knowledge, there is only one clinical study of the abscopal effect in metastatic melanoma patient treated with anti-PD-1 agents [10]. In this study, 12 patients with progressive metastatic melanoma disease on pembrolizumab or nivolumab were subsequently treated with radiation therapy. Three of which (25%) actually had distant response post-radiation.

With the increasing use of immunotherapy and reports documenting the abscopal effect, there has been a growing interest in studying their synergistic effect. A search with the keywords 'abscopal' and 'immunotherapy' on clinicaltrials.gov yielded nine clinical trials (Table 1). More studies are likely to come. Perhaps, with growing body of evidence, induction of abscopal effect would become an indication to offering radiation therapy in the near future. 

**Table 1:** Ongoing clinical trials The trials listed here were obtained from Clinicaltrials.gov with the keywords 'abscopal' and 'immunotherapy'.

Location	Title	Recruitment	Interventions	Phase	Enrollment
Ghent University Hospital (Belgium)	Trial of SBRT With Concurrent Ipilimumab in Metastatic Melanoma	Completed	Radiation: Stereotactic body radiotherapy (SBRT); Drug: Ipilimumab	1	13
Memorial Sloan Kettering (United States)	A Study of Durvalumab (Anti-PDL1) Plus Radiation Therapy for the Treatment of Solitary Bone Plasmacytoma	Suspended	Drug: Durvalumab; Radiation: Radiation therapy	1	20
Centre Hospitalier de l'Université de Montréal (Canada)	Immunotherapy and SBRT for Metastatic Head and Neck Carcinomas	Not yet recruiting	Radiation: SBRT; Drug: Durvalumab; Drug: Tremelimumab	1-2	45
University of California Davis Comprehensive Cancer Center (United States)	Phase I/II Trial of Epacadostat, Intralesional SD101, Radiotherapy in Patients With Lymphoma	Recruiting	Drug: Epacadostat; Drug: SD-101; Radiation: Radiotherapy	1-2	56
Gustave Roussy (France)	Atezolizumab With Stereotactic Ablative Radiotherapy in Patients With Metastatic Tumours	Recruiting	Drug: Anti-PD-L1 antibody Atezolizumab; Procedure: SABR	2	180
Zhongnan Hospital of Wuhan University (China)	A Study of SBRT in Combination With rhGM-CSF for Stage IV NSCLC Patients Who Failed in Second-line Chemotherapy	Recruiting	Radiation: Stereotactic body radiotherapy; Drug: rhGM-CSF	2	60
NYU Clinical Cancer Center (United States)	Phase II Randomized Trial of Ipilimumab Versus Ipilimumab and Radiotherapy in Metastatic Melanoma	Terminated	Drug: Ipilimumab; Other: Radiation Therapy and Ipilimumab	2	10
Brigham and Women's Hospital (United States)	Durvalumab, Tremelimumab + Radiotherapy in Gynecologic Cancer	Recruiting	Drug: Durvalumab; Drug: Tremelimumab; Radiation: Radiation Therapy	1	32
Dana Farber Cancer Institute (United States)	Targeting PD-1 Therapy Resistance With Focused High or High and Low Dose Radiation in SCCHN	Recruiting	Drug: Pembrolizumab; Radiation: Radiation	2	26

## Conclusions

As immunomodulatory therapy becomes the mainstay treatment for metastatic melanoma, reports of abscopal effects may become more common. The synergistic activity between radiotherapy and immunotherapy appears to be more frequent than originally thought. Case series with ipilimumab, and more recently a study with pembrolizumab and nivolumab are certainly encouraging. The quick time course and the extent of disease regression are what stand out in this patient.
